# Lifestyle treatment in women with polycystic ovary syndrome: predictors of weight loss and dropout

**DOI:** 10.1002/brb3.2621

**Published:** 2022-06-02

**Authors:** Geranne Jiskoot, Alexandra Dietz de Loos, Reinier Timman, Annemerle Beerthuizen, Joop Laven, Jan Busschbach

**Affiliations:** ^1^ Division of Reproductive Endocrinology and Infertility Department of Obstetrics and Gynecology Erasmus MC Rotterdam The Netherlands; ^2^ Department of Psychiatry Section Medical Psychology and Psychotherapy Erasmus MC Rotterdam The Netherlands

**Keywords:** cognitive behavioral therapy, depression, obesity

## Abstract

**Background:**

Polycystic ovary syndrome (PCOS) affects 5%–10% of women in their reproductive years. Most women with PCOS struggle with obesity during their entire life. Knowing which determinants contribute to a successful lifestyle change is important to optimize treatment options for women with PCOS.

**Objective:**

This analysis of secondary outcome measures aimed to determine factors of ≥5% weight loss and dropout in all arms of the study and separately in the lifestyle intervention (LI) and control (care as usual [CAU]) groups.

**Study design:**

Women diagnosed with PCOS (*N* = 183) and a Body Mass Index (BMI) above 25 kg/m^2^ were included. Participants were assigned to (1) 20 lifestyle sessions involving cognitive behavioral therapy (CBT), (2) 20 lifestyle sessions involving CBT with additional short message service (SMS), or (3) to control (CAU). A generalized linear regression was performed to identify determinants of ≥5% weight loss. Logistic regression was performed to identify determinants of dropout. All models were corrected by including baseline weight as a covariate.

**Results:**

LI (OR 4.906, *p* = .001) was associated with ≥5% weight loss, while higher depression scores (OR 0.549, *p* = .013) had a negative association. Restraint eating was a positive factor for ≥5% weight loss in LI but a negative in CAU. Higher baseline weight (OR 1.033, *p* = .006), LI with SMS (OR 4.424, *p* = .002), and higher levels of androstenedione (OR 1.167, *p* = .026) were associated with dropout.

**Conclusions:**

Depression and eating behavior were associated with ≥5% weight loss. Women with PCOS should be screened for depression and eating behavior before a LI.

## 1 INTRODUCTION

Polycystic ovary syndrome (PCOS) is a common endocrine disorder that affects 5%−10% of women in their reproductive years (Lim et al., 2012). Most women with PCOS struggle with obesity and weight gain during their entire life (Teede et al., 2013). Therefore, multicomponent (diet, exercise, and behavioral therapy) lifestyle interventions (LIs) are advised for women with PCOS (3). Most weight loss programs are effective in the short term; however, most of the initial weight loss is regained within 1 year (Brownell & Jeffery, 1987). Compared to one‐ or two‐component LIs, three‐component LIs have the largest effect to establish a long‐term weight loss in the general population (Dalle Grave et al., 2010). In the general population, substantial weight loss is difficult to achieve, and maintaining this weight loss is even a greater challenge (J. G. Thomas et al., 2014). In women with PCOS, weight loss might even be more difficult based on psychological factors like disordered eating, anxiety, mood disorders, and body image issues (Deeks et al., 2011; Pastore et al., 2011) and hormonal disturbances like hyperinsulinemia and hyperandrogenism (HA) affecting abdominal fat deposition (Moran et al., 2010) or appetite regulation (Moran et al., 2004). Primarily, depression scores are significantly higher in women with PCOS, compared to women without PCOS (Deeks et al., 2011; Moran et al., 2010). The prevalence of depression is almost 37%, compared to 14% in controls (Pastore et al., 2011). In the general population, there is a bidirectional association between depression and obesity (Jiskoot et al., 2017). In women with PCOS, the results are inconclusive: Some authors concluded that ody Mass ndxndx BMI and depression are associated, while others suggested the opposite. When women with PCOS were matched on Body Mass Index BMI, they still had higher odds for depressive and anxiety symptoms (Pastore et al., 2011). Despite the evidence that women with PCOS have increased odds for depression and anxiety, there is no evidence supporting a single etiology for this increased prevalence of depression and anxiety (Beck et al., 1996).

It would be helpful to identify pretreatment‐related factors associated with successful weight loss to identify women who may benefit from such a lifestyle program or who need alternative support to achieve weight loss. This is especially because treatment adherence is low and noncompletion rates are high (Moroshko et al., 2011). In the general population, successful weight loss was linked to demographic, behavioral, psychological, social, and physical environmental determinants (Jiandani et al., 2016; Mitchell et al., 2017). In a recent meta‐analysis, it was found that only self‐monitoring of weight or food and eating behaviors, such as the ability to control portions, were strong predictors of weight loss. Successful weight loss was neither predicted by age, gender, and socioeconomic status, nor were high depression scores, low quality of life (QoL), and motivation involved in weight loss (Varkevisser et al., 2019). In addition, weight loss during the first 3 months of a lifestyle program seems to predict weight loss at the end of the program (James et al., 2018; D. M. Thomas et al., 2015). In a lifestyle program for infertile women, higher external eating behavior scores and not receiving previous support from a dietician were associated with success (Karsten et al., 2019). In women with PCOS, ≥5% weight loss at 2 months was associated with better QoL scores related to infertility. Lower age and a higher attendance rate were associated with ≥5% weight loss. In this study, no relationships were found between demographic, anthropometric, clinical, or hormonal factors and weight loss in women with PCOS (Moran et al., 2019).

A systematic review revealed that only four out of 15 LIs for women with infertility reported baseline characteristics that were associated with dropout (Mutsaerts et al., 2013). A small study in women with PCOS found higher free testosterone and total testosterone levels in women who dropped out from a LI (Kuchenbecker et al., 2011). Moran et al. (2019) analyzed data from four different LIs to identify participant and intervention characteristics for dropout in women with PCOS. A dropout rate of 47.1% was found, and most of the participants dropped out before 8 weeks. Dropout was associated with lower fasting glucose levels, better baseline QoL related to body hair, lower QoL related to infertility, and study attendance. In addition, baseline depression scores tended to be higher in women who dropped out.

Based on previous research, it is believed that LIs with a behavioral component can further improve attrition and weight loss (Moran et al., 2019). Therefore, we want to identify those women who are most likely to succeed and will benefit most from altering their lifestyle through a three‐component intervention. The objective of the present study was to investigate demographical, PCOS characteristics, psychological and behavioral related determinants that contributed to a ≥5% weight and dropout in all arms of the study and separately in LI and care as usual (CAU). Knowing which patient‐related determinants contribute to a successful lifestyle change is important to find out what is most effective for whom and to optimize treatment options for women with PCOS.

## 2 MATERIALS AND METHODS

### 2.1 Study design

This study used data from a randomized controlled trial in 183 women with PCOS. Participants were randomized into either (1) 20 group sessions of cognitive behavioral therapy (CBT) for 1 year combined with a healthy diet and exercise, (2) 20 group sessions of CBT for 1 year combined with a healthy diet and exercise with an additional 9 months of electronic feedback through short message service (SMS), or (3) the control group who received usual care that constituted advice to lose weight. The primary outcome of the intervention was weight loss, and these results have been described previously (Jiskoot et al., 2020). In summary, during the study, 21.8% of the women in the CAU group achieved 5% weight loss, compared to 52.8% of the women in the LI without SMS group, and 85.7% in the LI with SMS group (OR 7.0, *p* < .001). There were no significant differences in dropout rates between the three arms of the study: 60.0% in CAU, 73.4% in LI without SMS, and 57.2% in LI with SMS. The overall dropout rate was 116/183 = 63.4% (Jiskoot et al., 2020). The RCT was approved by the Medical Research Ethics Committee of the Erasmus MC in Rotterdam, reference number MEC 2008—337, and registered at the Dutch trial register by number NTR2450.

### 2.2 Participants

Women were eligible if they were diagnosed with PCOS according to the Rotterdam 2003 consensus criteria, had a BMI above 25 kg/m^2^, were between 18 and 38 years old and were trying to become pregnant. Women with inadequate command of the Dutch language, severe mental illness, obesity with another somatic cause, ovarian tumors that lead to androgen excess, adrenal diseases, or having malformations of their internal genitalia, or women who were pregnant, were not eligible for the study.

All participants attended the outpatient clinic at baseline and 3, 6, 9, and 12 months for standardized screening, and all outcome measures were assessed. This screening included a family and reproductive history and anthropomorphometric and ultrasonographic assessments. Participants also completed several psychological questionnaires at these time points.

### 2.3 LI and CAU

The LI consisted of 20 group sessions of 2.5 h, of which the first 1.5 h of every group session were supervised by a psychologist and dietician. The last hour of the group sessions was supervised by two physical therapists. The aim of the LI was a healthy weight loss of 5% to 10% through CBT, healthy dietary habits, physical activity, and activating social support. During the CBT sessions, different principles and techniques were discussed like self‐monitoring and goal setting. The development of new coping skills to handle or prevent relapses played an important role in the sessions and homework assignments (Jiskoot et al., 2017). In addition, thought records were used for cognitive restructuring (Beck, Steer, Ball, & Ranieri, 1996). A normo‐caloric diet was advised based on the Dutch Food Guide (Beck, Steer, & Brown, 1996). Participants did not receive any caloric restriction or lists of prescribed foods in set quantities. Also, techniques of intuitive eating, such as eating when hungry and stopping with eating when satisfied, were discussed during the sessions. During the exercise sessions, different kinds of sports were offered. Participants were motivated to increase their daily physical activity and to find a sport they enjoy doing. More details about the intervention can be found in the study protocol (Jiskoot et al., 2017). After 3 months, half of the LI participants received additional support by tailored SMS messages via mobile phone (LI with SMS). Participants sent weekly self‐monitored information regarding their diet, physical activity, and emotions by SMS to the psychologist. They received feedback and two messages per week addressing healthy lifestyle habits. Participants in the CAU (control) were advised to achieve weight loss by publicly available methods (e.g., visit a dietician or membership with a local gym). In addition, they had consultations with their treating physician during the study appointments at baseline and at 3, 6, 9, and 12 months.

### 2.4 Outcomes

This analysis of secondary outcome measures aimed to determine factors of ≥5% weight loss and dropout in all arms of the study and separately in the LI and CAU. Demographic and PCOS characteristics, as well as psychological data, were all assessed at baseline and categorized into several domains, namely:

Demographic characteristics: age, ethnicity, education.
Lifestyle characteristics: alcohol use and smoking at baseline.
PCOS characteristics: polycystic ovarian morphology, ovulatory dysfunction, amenorrhea, oligomenorrhea, HA, clinical HA (modified Ferriman Gallwey score ≥5), and biochemical HA (Free Androgen Index [FAI] > 2.9).
Infertility characteristics: duration of infertility in months, null parity.
Anthropometric and weight characteristics: weight (kg), BMI in kg/m^2^, waist and hip circumference in centimeters, and the waist–hip ratio at baseline.
Metabolic characteristics: glucose, insulin, and cortisol were collected between 8:00 and 11:00 a.m. after overnight fasting.
Androgens: serum testosterone, androstenedione, dehydroepiandrosterone, and sex hormone‐binding globulin.
Study arms: LI, CAU, and separately the LI without additional SMS versus LI with additional SMS.
Psychological characteristics: depression, self‐esteem, body image, eating psychopathology, emotional eating, external eating, the tendency for dietary restraint, and QoL. Depression was measured with the Beck Depression Inventory‐II (Beck, Steer, Ball, & Ranieri, 1996; Beck, Steer, & Brown, 1996), where a higher score denotes more severe depression. Self‐esteem and self‐acceptance are measured by the Rosenberg Self Esteem Scale (Franck et al., 2008; Rosenberg, 2015), where a higher score indicates higher levels of self‐esteem. Body image was measured by the brief version of the Fear of Negative Appearance Evaluation Scale (Lundgren et al., 2004), whereby a higher score indicates more fear of negative evaluation by others. Eating psychopathology was measured by the Eating Disorder Examination Questionnaire (Fairburn & Beglin, 1994, 2008). This questionnaire consists of five subscales: concerns about shape, weight, and eating, in addition to restrained and binge eating. A higher score indicates more severe eating psychopathology. The Dutch Eating Behavior Questionnaire (DEBQ; Van Strien et al., 1986) is used to assess eating in response to negative emotions (subscale emotional eating and subscale diffuse emotions), eating in response to the sight or smell of food (subscale external eating), and eating less than desired to lose or maintain body weight (subscale restraint eating). A higher score indicates a higher degree of the relevant eating behavior. QoL is measured by the QoL Short Form 36 (Aaronson et al., 1998) and consists of eight dimensions. The eight dimensions can be grouped into Physical and Mental Component Summary scores (Ware et al., 2001).

### 2.5 Statistical analysis

We made a preselection of potential predictors based on a literature search to limit the possibility of overfitting the prediction model. All predictor variables were standardized for ease of interpretation. As described in the study protocol, the LI without SMS and LI with additional SMS were pooled to examine the effect of LI, compared to CAU. A generalized linear regression (GENLIN) was performed to identify determinants of ≥5% weight loss. This statistical model can efficiently deal with missing data and unbalanced time points (Little & Rubin, 1987; Roderick & Donald, 1986). This analysis included two levels: the patients constituted the upper level, and their baseline measures constituted the lower level. Study group, logarithmic time, and interactions were included as independent variables. Logistic regression was performed to identify variables that were associated with dropout. All models were corrected by including baseline weight as a covariate. First, we performed univariate models, and predictors with a significance of < 0.20 were entered into a multivariate model. In a backward elimination procedure, predictor variables that did not (significantly *p* < .05) contribute to the dependent measure were removed from the model one by one. All analyses were performed with IBM Corp (Released 2017. IBM SPSS Statistics for Windows, Version 25.0.).

## 3 RESULTS

**FIGURE 1:**
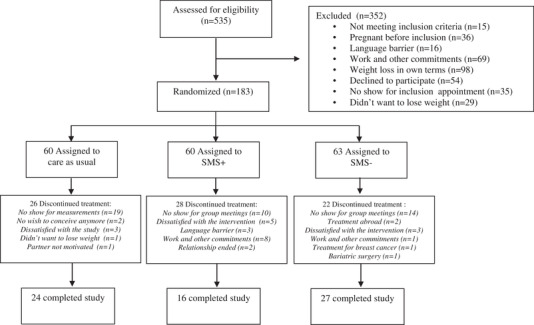
CONSORT flowchart

Between August 2, 2010, and March 11, 2016, 535 eligible women were asked to participate, and 209 provided written informed consent, of whom 26 were included in a pilot study. At baseline, 63 participants were randomized to LI without SMS, 60 to LI with SMS, and 60 to CAU (Figure 1). A total of 490 measurements belonging to 183 participants were included in the analyses. The baseline characteristics of the participants are described in Table 1. The mean age was 29.1 (±4.4) years, and the average infertility duration was 33.5 (±31.7) months. Most participants (36.1%) had intermediate levels of education and were nulliparous (76.0%). The present analysis confirmed the findings published before that the LI intervention had a significant effect on weight loss and dropout (Jiskoot et al., 2020). Below, we present the effects of the baseline predictors.

**TABLE 1:** Baseline characteristics

	Care as usual (CAU) (*n* = 60)	Lifestyle intervention (LI) without short message service (SMS; *n* = 63)	LI with SMS (*n* = 60)	Total (*n* = 183)
	Mean (SD)	Mean (SD)	Mean (SD)	Mean (SD)
Age (year)	28.5 (4.3)	29.9 (4.3)	28.7 (4.6)	29.1 (4.4)
Weight (kg)	89.5 (15.8)	91.7 (14.3)	96.4 (14.6)	92.5 (15.1)
BMI (kg/m^2^)	32.7 (5.1)	34.0 (4.4)	34.7 (4.9)	33.7 (4.9)
Time attempting to conceive (months)	35.8 (30.8)	38.9 (36.7)	25.1 (25.2)	33.5 (31.7)

	*n* (%)	*n* (%)	*n* (%)	*n* (%)
Caucasian	19 (31.7)	20 (31.7)	26 (43.3)	65 (35.5)
Smoking	15 (25.0)	12 (19.0)	13 (21.7)	40 (21.9)
Alcohol consumption	21 (35.0)	15 (23.8)	12 (20.0)	48 (26.2)
Nulliparous	44 (73.3)	48 (76.2)	47 (78.3)	139 (76.0)
Education				
* Low*	7 (11.7)	4 (6.3)	5 (8.3)	16 (8.7)
* Intermediate*	17 (28.3)	24 (38.1)	25 (41.7)	66 (36.1)
* High*	6 (10.0)	16 (25.4)	11 (18.3)	33 (18.0)
* Missing*	30 (50.0)	19 (30.2)	19 (31.7)	68 (37.2)
Dropout	36 (60.0)	36 (57.1)	44 (73.3)	116 (63.4)

### 3.1 Determinants of ≥5% weight loss

In the univariate models (Table 2), participating in the lifestyle treatment (odds ratio [OR] 1.805, *p* = .008), additional SMS (OR 1.407, *p* = .077), presence of HA (OR 0.736, *p* = .105), presence of oligomenorrhea (OR 0.778, *p* = .181), insulin (OR 0.612, *p* = .091), cortisol (OR 0.785, *p* = .199), depression (OR 0.653, *p* = .062), physical QoL (OR 1.542, *p* = .081) and mental QoL (OR 1.478, *p* = .114) had *p*s < .20 and were therefore included in the multivariable model. The multivariable mixed‐effect logistic regression model showed that participating in the lifestyle treatment (OR 4.906, CI 1.946–12.366, *p* = .001) was significantly associated with a higher proportion to achieve ≥5% weight loss. While, more depressive symptoms (OR 0.549, CI 0.34–0.88, *p* = .013) were significantly associated with a lower proportion to achieve ≥5% weight loss.

**TABLE 2:** Univariate model: Determinants of ≥5% weight loss and dropout at 12 months

	≥5% weight loss		Dropout	
Determinants	OR (95% CI) univariate	*p*‐value	OR (95% CI) univariate	*p*‐value
Study arm (CAU vs. LI)	1.805 (1.169–2.786)	*.008*	0.476 (0.237–0.918)	*.027*
SMS+ versus SMS–	1.407 (0.964–2.055)	*.077*	1.570 (0.812–3.035)	*.180*
Age	1.228 (0.830–1.816)	.304	0.997 (0.928–1.072)	.946
Smoking	0.825 (0.568–1.197)	.311	0.504 (0.215–1.184)	*.116*
Alcohol intake	0.831 (0.557–1.240)	.364	1.951 (0.973–3.911)	*.060*
Months attempting to conceive	0.838 (0.454–1.545)	.571	1.003 (0.992–1.015)	.572
Multiparous	1.212 (0.816–1.801)	.340	0.675 (0.303–1.504)	.336
OD	0.880 (0.576–1.344)	.554	0.608 (0.129–2.872)	.530
PCOM	0.947 (0.890–1.007)	.084	0.826 (0.073–9.402)	.877
Oligomenorrhea	0.778 (0.539–1.123)	*.181*	0.659 (0.309–1.406)	.659
Amenorrhea	1.232 (0.864–1.756)	.248	1.493 (0.654–3.410)	.341
HA	0.736 (0.508–1.066)	*.105*	1.558 (0.764–3.177)	.222
Biochemical HA	0.847 (0.601–1.194)	.343	1.032 (0.974–1.094)	.288
Clinical HA	0.775 (0.504–1.190)	.244	0.809 (0.409–1.602)	.543
Glucose	0.972 (0.851–1.111)	.677	0.855 (0.488–1.498)	.584
Insulin	0.612 (0.346–1.082)	*.091*	1.003 (0.999–1.007)	*.150*
Testosterone	0.976 (0.679–1.403)	.895	1.153 (0.815–1.631)	.421
Cortisol	0.785 (0.543–1.135)	*.199*	1.000 (0.998–1.003)	.784
SHBG	0.946 (0.720–1.244)	.692	1.004 (0.984–1.025)	.667
DHEA	0.821 (0.505–1.335)	.426	0.998 (0.988–1.008)	.673
Androstenedione	0.832 (0.553–1.250)	.375	1.095 (0.971–1.235)	*.139*
Depression (BDI‐II)	0.653 (0.417–1.022)	*.062*	1.011 (0.978–1.045)	.530
Body image (FNAE)	0.788 (0.544–1.140)	.206	1.015 (0.971–1.062)	.504
Self‐esteem (RSE)	1.275 (0.819–1.985)	.283	0.974 (0.917–1.033)	.375
Eating psychopathology (EDEQ)	1.030 (0.714–1.487)	.873	1.123 (0.887–1.422)	.334
DEBQ Subscale Diffuse emotions	1.302 (0.849–1.996)	.226	0.981 (0.703–1.370)	.911
DEBQ Subscale Emotional eating	1.088 (0.724–1.635)	.684	1.100 (0.768–1.575)	.603
DEBQ Subscale Restraint	1.080 (0.707–1.649)	.721	0.951 (0.582–1.554)	.841
DEBQ Subscale External eating	1.242 (0.795–1.939)	.341	0.872 (0.476–1.594)	.655
Quality of life (QoL; SF36) physical	1.542 (0.948–2.508)	*.081*	0.996 (0.976–1.016)	.702
QoL (SF36) mental	1.478 (0.910–2.399)	*.114*	0.995 (0.978–1.013)	.619

*Note*: All models were corrected for baseline weight.

Abbreviations: BDI‐II, Beck Depression Inventory‐II; DEBQ, Dutch Eating Behavior Questionnaire; DHEA, dehydroepiandrosterone; EDEQ, Eating Disorder Examination Questionnaire; FNAE, Fear of Negative Appearance Evaluation Scale; HA, hyperandrogenism; OD, ovulatory dysfunction; PCOM, polycystic ovarian morphology; RSE, Rosenberg Self Esteem scale; SF36, Short Form 36; SHBG, sex hormone‐binding globulin.

### 3.2 Determinants of ≥5% weight loss in LI and CAU

Determinants that were associated with ≥5% weight loss were separately tested in LI and CAU. The multivariable mixed‐effect logistic regression model showed that in LI, higher baseline weight (OR 0.466, *p* = .003) and worse body image (OR 0.233, *p* < .001) were associated with a lower proportion to achieve ≥5% weight loss. A higher tendency for restraint eating (OR 5.164, *p* = .005), a higher tendency for external eating (OR 3.094, *p* = .001), and the presence of amenorrhea (OR 7.416, *p* = .006) were associated with a higher proportion to achieve ≥5% weight loss. In CAU, a higher baseline weight (OR 1.915, *p* = .026) was associated with a higher proportion to achieve ≥5% weight loss, while a higher tendency for restraint eating (OR 0.587, *p* < .001) was associated with a lower proportion to achieve ≥5% weight loss (Table 3).

**TABLE 3:** Multivariate model: Determinants of ≥5% weight loss in lifestyle and CAU

Lifestyle			CAU		
Determinants	OR (95% CI)	*p*‐value	Determinants	OR (95% CI)	*p‐*value
Baseline weight	0.466 (0.283–0.769)	.003	Baseline weight	1.915 (1.079–3.399)	.026
Body image	0.230 (0.112–0.474)	< .001	Restraint eating	0.587 (0.437–0.790)	< .001
Restraint eating	5.164 (1.661–16.048)	.005			
External eating	3.094 (1.615–5.925)	.001			
Amenorrhea	7.416 (1.768–31.111)	.006			

### 3.3 Determinants of dropout

A dropout rate of 36/60 (60.0%) was observed in CAU, 36/63 (57.1%) in the LI without SMS, and 44/60 (73.3%) in the LI with SMS. The overall dropout rate was 116/183 (63.4%). In the univariate regression models, participating in the lifestyle group (OR 0.446, *p* = .027), additional SMS (OR 1.570, *p* = .180), smoking (OR 0.504, *p* = .116), drinking alcohol (OR 1.951, *p* = .060), insulin (OR 1.003, *p *= .150), and androstenedione (OR 1.095, *p* = .139) had *p*s < .20 and were therefore included in the multivariable model (Table 2). The multivariable regression models showed that higher baseline weight (OR 1.033, *p* = .006), participation in LI with SMS (OR 4.424, *p* = .002), and higher levels of androstenedione (OR 1.167, *p* = .026) were significantly associated with higher odds of dropout. Participation in the control group (OR 0.173, *p* < .001) and smoking (OR 0.349, *p* = .031) were associated with lower odds of dropout (Table 4).

**TABLE 4:** Multivariate model: Determinants for dropout

Determinants	*β*	OR (95% CI) univariate	*p*‐value
Baseline weight	0.032	1.033 (1.009–1.057)	.006
Study arm (LI vs. CAU)	–1.752	0.173 (0.066–0.454)	< .001
SMS+ versus SMS–	1.487	4.424 (1.732–11.298)	.002
Smoking	–1.052	0.349 (0.134–0.907)	.031
Androstenedione	0.154	1.167 (1.019 1.336)	.026

### 3.4 Determinants of dropout in LI and CAU

Determinants that were associated with dropout were separately tested in LI and CAU. The multivariable regression models showed that in LI, higher baseline weight (OR 1.04, *p *= .007) and additional SMS (OR 4.31, *p *= .002) were associated with higher odds of dropout, while in CAU, no significant predictors for dropout were found.

## 4 COMMENT

This study investigated patient‐related determinants that predicted weight loss and dropout during an RCT of a three‐component CBT LI, compared to CAU in women with PCOS. We observed that participating in the LI was associated with a higher proportion of ≥5% weight loss, and higher depressive symptoms were associated with a lower proportion of ≥5% weight loss. Logistic regression showed that higher baseline weight, participation in LI without SMS, and higher androstenedione levels resulted in a higher proportion of dropouts.

We found that especially higher depression scores were associated with a lower proportion to achieve ≥5% weight loss. In the general population, there is a negative bidirectional relationship between obesity and depression. Obesity was found to increase the risk of depression, but depression was also found to increase the risk of developing obesity (Luppino et al., 2010). A large meta‐analysis tested the effects of weight loss on depression scores and found that lifestyle modification and not the weight loss itself was associated with significant reductions in depression scores (Fabricatore et al., 2011). In women with PCOS, the same association between LI and improvements in depression scores was found (Jiskoot et al., 2020; Thomson et al., 2010). Higher depression scores were also associated with dropout during lifestyle treatment in women with PCOS (Moran et al., 2019). Therefore, others advised additional psychological treatment for depressed participants before entering a LI (McLean et al., 2016).

We found differences in baseline characteristics between women who were successful in LI and CAU. This implies that different characteristics are involved to achieve ≥5% weight loss based on the type of intervention women received. In CAU, higher baseline weight and higher scores for restraint eating were associated with a lower proportion to achieve ≥5% weight loss. While in LI, women with higher baseline weight and worse body image were less able to achieve ≥5% weight loss and had also higher scores for restraint eating and higher scores for external eating. Moreover, the presence of amenorrhea was significantly associated with a higher proportion to achieve ≥ 5% weight loss. This suggests that disordered eating behavior, especially restraint eating, played an important role in the pathway of success in both groups. Disordered eating includes the full spectrum of eating‐related problems like emotional eating, restrained eating, and episodes of binge eating (American Psychiatric Association, 2006). Restrained eating refers to “chronic dieting” or intentional restriction of food intake to influence body weight, often interrupted with episodes of overeating. After these periods of overeating or eating “forbidden” foods, restraint eaters tend to consume more in general (Lowe & Thomas, 2009; Stroebe, 2008). Higher scores for restraint eating resulted in a lower chance to achieve weight loss in CAU, while higher scores for restraint eating resulted in a higher chance for ≥5% weight loss in LI.

In CAU, women were advised to lose weight by publicly available services like following a popular diet on the Internet. Most of the available diets advocate dietary restraint by forbidding certain types of foods or food groups, such as bread or carbohydrates. There seems to be a relationship between restricted diets and the chances to develop disordered eating behavior. In several studies, restricted diets were the strongest risk factor for the development of disordered eating (Watson, 2011; Watson et al., 2010) and weight gain (Langeveld & DeVries, 2015). In LI, CBT was used as a technique for challenging and changing dysfunctional eating and body‐related beliefs and schemas to develop and maintain a healthier eating pattern (Werrij et al., 2009). In the general population, CBT seems effective to develop healthy eating behavior (Werrij et al., 2009), especially in women with bulimia nervosa and binge eating disorder (Linardon et al., 2017). Therefore, CBT seems to be the driving factor in achieving successful weight loss by changing dysfunctional eating patterns. Indeed, women with higher scores for restraint eating who participated in the LI group seem to have higher odds to lose weight based on the CBT component. This finding was also seen in another long‐term CBT weight‐loss program where higher scores for dietary restraint were associated with more weight loss (Volger et al., 2013). Based on these findings, it seems important to screen women with PCOS for disordered eating before they attempt weight loss.

Future research should examine if the current three‐component LI should be altered for women who are not successful in achieving a ≥5% weight loss. A recent study examined the effects of additional support (one individual meeting and two phone calls) for participants who were not successful at Week 4 of a lifestyle program. The additional support resulted in more weight loss and better adherence to the lifestyle program (Unick et al., 2016). It could also be hypothesized that women should be selected based on their baseline characteristics (e.g., depression or eating behavior) before entering a three‐component LI. Therefore, we will examine the effects of this three‐component LI, compared to gastric bypass surgery in women with PCOS, especially, to examine which treatment works best for this large and diverse group of women.

A strength of the current study is that we examined psychological determinants and PCOS characteristics in the relationship between weight loss and dropout. We found that women with higher levels of androstenedione were more likely to drop out from the study. Moreover, androstenedione was highly correlated with testosterone, the FAI, and HA by Pearson's correlation analysis (data not shown). Based on previous work, elevated serum androstenedione seems to be associated with a more severe PCOS phenotype (Georgopoulos et al., 2014). During a 6‐month diet intervention with 1200−1400 kcal per day, androstenedione was the only significant predictor for the complete “disappearance” of PCOS (Pasquali et al., 2011). We could only establish that androstenedione was involved in dropout and not in whether one achieved considerable weight loss. Nevertheless, we think that success and dropout belong to the same phenomenon and therefore argue that androstenedione is indeed an important clinical marker for weight loss in women with PCOS.

A limitation of our study is the high discontinuation rate we observed in all three arms of the study. Compliance and dropout are the most difficult aspects of any weight‐reduction intervention, especially in programs that last over 42 weeks (Mutsaerts et al., 2013). About one‐third drop out from general weight loss programs (Mutsaerts et al., 2013), and this can even increase up to 80% (Davis & Addis, 1999). We expected high dropout rates expected in this 1‐year intervention; therefore, a statistical method was chosen that could include all available data even if participants dropped out during the study period. An important factor that could be linked to dropout is social support during lifestyle treatment. Other lifestyle programs found that social support and sabotage from friends and family were associated with weight loss in women during lifestyle treatment (Kiernan et al., 2012). It is unclear in our study whether social support or other factors like the intensity of the program or spontaneous pregnancies, were associated with dropout. We tried to contact women who dropped out of the intervention, but most of them were not willing to provide more information about their reasons for ending the study. However, having data on the ones that dropped out and taking into account a large number of dropouts, one might consider this as well as a strength of this study.

### 4.1 Conclusion

Participation in the lifestyle treatment resulted in a higher proportion to achieve ≥5% weight loss, while more depressive symptoms resulted in a lower proportion. Dropout seems to be related to baseline weight and higher levels of androstenedione. Women with these characteristics should be more encouraged to complete a LI. Hence, a three‐component LI based on CBT can be successful in improving mood in women with PCOS who are overweight or obese and attempting to become pregnant. Women with PCOS should be screened for depression and eating behavior before entering an LI to improve weight loss.

## CONFLICT OF INTEREST

Geranne Jiskoot, Alexandra Dietz de Loos, Reinier Timman, Annemerle Beerthuizen, Jan Busschbach have nothing to declare. Joop Laven has received unrestricted research grants from Ansh Labs, Ferring and Roche Diagnostics. He received consultancy fees from the following companies: Ansh Labs, Ferring, Roche Diagnostics, and Titus Healthcare.

## AUTHOR CONTRIBUTION

Geranne Jiskoot, Alexandra Dietz de Loos, Reinier Timman, Annemerle Beerthuizen, Joop Laven, and Jan Busschbach made substantial contributions to the conceptualization, study design, execution, implementation, and writing of this paper. Geranne Jiskoot and Reinier Timman performed formal statistical analyses.

### PEER REVIEW

The peer review history for this article is available at https://publons.com/publon/10.1002/brb3.2621.

## Data Availability

All of the individual participant data collected during the trial, after de‐identification is available. Proposals should be directed to the first author. To gain access, data requestors will need to sign a data access agreement.
